# Multilevel analysis of social determinants of advanced stage colorectal cancer diagnosis

**DOI:** 10.1038/s41598-024-60449-0

**Published:** 2024-04-26

**Authors:** Amanda Almeida Gomes Dantas, Nayara Priscila Dantas de Oliveira, Guilherme Augusto Barcello Costa, Luís Felipe Leite Martins, Jonas Eduardo Monteiro dos Santos, Arn Migowski, Marianna de Camargo Cancela, Dyego Leandro Bezerra de Souza

**Affiliations:** 1 Graduate Program in Health Sciences, Federal University of Rio Grande do Norte – UFRN, Natal, RN Brazil; 2 Department of Physical Therapy, University of Pernambuco – UPE, Petrolina, PE Brazil; 3 Graduate Program in Oncology, Research and Innovation Coordination, National Cancer Institute (INCA), Ministry of Health, Rio de Janeiro, RJ Brazil; 4 Surveillance and Situation Analysis Division, Prevention and Surveillance Coordination (CONPREV), National Cancer Institute (INCA), Ministry of Health, Rio de Janeiro, RJ Brazil; 5 Division of Clinical Research and Technological Development, Research and Innovation Coordination, National Cancer Institute (INCA), Ministry of Health, Rio de Janeiro, RJ Brazil; 6 Professional Master’s Program in Health Technology Assessment, Education and Research Coordination, National Institute of Cardiology (INC), Ministry of Health, Rio de Janeiro, RJ Brazil; 7 Graduate Program in Public Health, Federal University of Rio Grande do Norte – UFRN, Natal, RN Brazil; 8 Methodology, Methods, Models and Results in Health and Social Sciences Research Group (M3O), Faculty of Health Sciences and Well-Being. Health and Social Care Research Center (CESS), University of Vic-Central University of Catalonia (UVic-UCC), Vic, Spain; 9 Public Health Department, Graduate Program in Public Health, Federal University of Rio Grande do Norte, 1787 Senador Salgado Filho Ave., Lagoa Nova, Natal, RN 59010-000 Brazil

**Keywords:** Colorectal cancer, Diagnosis, Social determinants of health, Health inequalities, Screening, Cancer epidemiology, Gastrointestinal cancer

## Abstract

The advanced stage at diagnosis of colorectal cancer (CRC) may be related to individual factors, socioeconomic conditions, and healthcare service availability. The objective of the study was to analyze the prevalence of advanced stage CRC at the time of diagnosis and its association with individual, contextual, socioeconomic, and healthcare service indicators. An observational, cross-sectional study was conducted, analyzing cases of malignant neoplasms of the colon and rectum in individuals of both sexes, aged between 18 and 99 years, diagnosed between 2010 and 2019 in Brazil (n = 69,047). Data were collected from the Hospital Cancer Registry (HCR), Atlas of Human Development in Brazil, and from the National Registry of Health Institutions (NRHI). A Multilevel Poisson Regression model with random intercept was used. The prevalence of advanced stage CRC at diagnosis was 65.6%. Advanced stage was associated with older age groups prevalence ratio (PR) 4.40 and younger age groups (PR 1.84), low Human Development Index (HDI) (PR 1.22), and low density of family health strategy teams (PR 1.10). The study highlights the unequal distribution of social determinants of health in the diagnosis CRC in Brazil, revealing the need to evaluate and redirect public policies aimed at improving early detection and prevention of CRC in the country.

## Introduction

Colorectal cancer (CRC) is the third most common malignant neoplasm and the second leading cause of cancer-related deaths worldwide, accounting for 10% to 12% of all cancer cases^1,2^. This condition poses a significant global burden due to its complications, mortality, adverse effects of treatment, healthcare utilization, and direct and indirect costs^3^.

In 2020, 1.93 million cases of CRC and 0.9 million deaths were reported worldwide^4^. In Brazil, according to the National Cancer Institute (INCA), for each year of the 2023–2025 triennium, an estimated 45,630 cases were projected, corresponding to a risk of 21.10 cases per 100,000 inhabitants. This represents an assessed risk of 20.78 new cases per 100,000 men and 21.41 per 100,000 women ^5^.

Due to the lack of symptoms during the early stages of CRC, nearly half of the patients are diagnosed at advanced stages, which impacts their survival^2^. The 5 year survival rates for those with early-stage disease (Stage I and II) approach 90%, while for those diagnosed at advanced stages (Stage III and IV) the survival rate is around 15% in the United States, according to a population-based study conducted with data from the period 2001–2019^6^. At this stage, treatment often becomes palliative, and the financial burdens are greater for healthcare systems^7^.

The morbidity and mortality, and increasing incidence of CRC are a reflection of the social determinants of individual and population health conditions, including biological, lifestyle, dietary, socioeconomic, and environmental factors, as well as healthcare service provision^8,9^.

The increase in CRC mortality rates observed in numerous countries in Latin America, the Caribbean, and Asia^10,11^ may reflect limited healthcare infrastructure and less access to early detection and treatment^12^, as it is associated with the stage of cancer at the time of diagnosis^13,14^.

Currently, there are no studies in our country that specifically investigate the relationship between individual, population, and healthcare service factors with the stage at diagnosis of CRC. The Brazilian territory exhibits economic, social, and demographic inequalities that impact the distribution and access to healthcare services^15^. This study aims to analyze the prevalence of advanced stage CRC at the time of diagnosis and its association with clinical, socioeconomic, and healthcare service factors in Brazil.

## Results

Between 2010 and 2019, 145.627 cases of CRC were registered in individuals aged 18 to 99 years, 37,633 (25.3%) in early stage, 67,767 (46.51%) in advanced stage, and 40,297 (27.66%) with missing staging data. In Table 1, a descriptive analysis of the individual data of present and absent staging for CRC was presented.

**Table 1:** Descriptive analysis for present and absent staging data at the diagnosis of colon and rectal cancer, according to individual characteristics.

	Known stage		Stage missing	
	n	%	n	%
Sex				
Female	51.592	71.4	20.679	28.6
Male	53.738	73.3	19.618	26.7
Age group				
18–49 years old	9.815	37.1	16.686	62.9
50–59 years old	25.500	73.7	9.080	26.3
60–69 years old	35.064	83.4	6.973	16.6
70 years or older	34.951	82.9	7.558	17.1
Race				
White	25.413	63.5	14.610	36.5
Non white	36.556	69.7	15.854	30.3
No Information	43.361	81.5	9.833	18.5
Primary tumor location				
Right	22.656	74.3	7.830	25.7
Left	82.674	85.9	32.467	14.1
Education				
None/Incomplete fundamental education	42.741	76.5	13.083	23.5
Fundamental education	17.661	74.8	5.928	25.2
Secondary education/ incomplete	15.703	68.1	7.354	31.9
Undergraduate education	6.905	72.0	2.681	28.0
No information	22.320	66.5	11.251	33.5
Marital status				
Unmarried	23.069	66.4	11.653	33.6
Married	33.400	67.2	16.327	32.8
No information	48.861	79.8	12.317	20.2
Source of referral				
Public (SUS)	44.829	65.6	23.505	34.4
Private/health insurance	9.779	67.8	4.644	32.2
No information	50.722	80.6	12.148	19.4

Brazil, by place residence (n = 145,627).

A bivariate and multivariate analysis of the missing data was conducted before the final model was built with the exclusion of these data. The bivariate regression shows that the absence of data is associated with individual, contextual, and healthcare service factors, as demonstrated in Table S1. Multilevel analysis allows for stratifying groups of variables according to aggregated levels. Level 1 includes cases reported to the RHC individual variables, while at Level 2, socioeconomic indicators and the provision of health services by state (FU) are considered. This analysis demonstrates the association of the lack of staging with individual factors such as age groups, as well as with contextual factors such as the density of oncologists and proctologists and specialized oncology services (Table S2). It is important to highlight that it was decided to exclude all cases of CRC from the Cancer Hospital Registry (RHC) of the state of São Paulo (n = 42,260) because individual socioeconomic information for the cancer cases was not available. A total of 103,367 CRC cases were included in these analyses.

**Figure 1:**
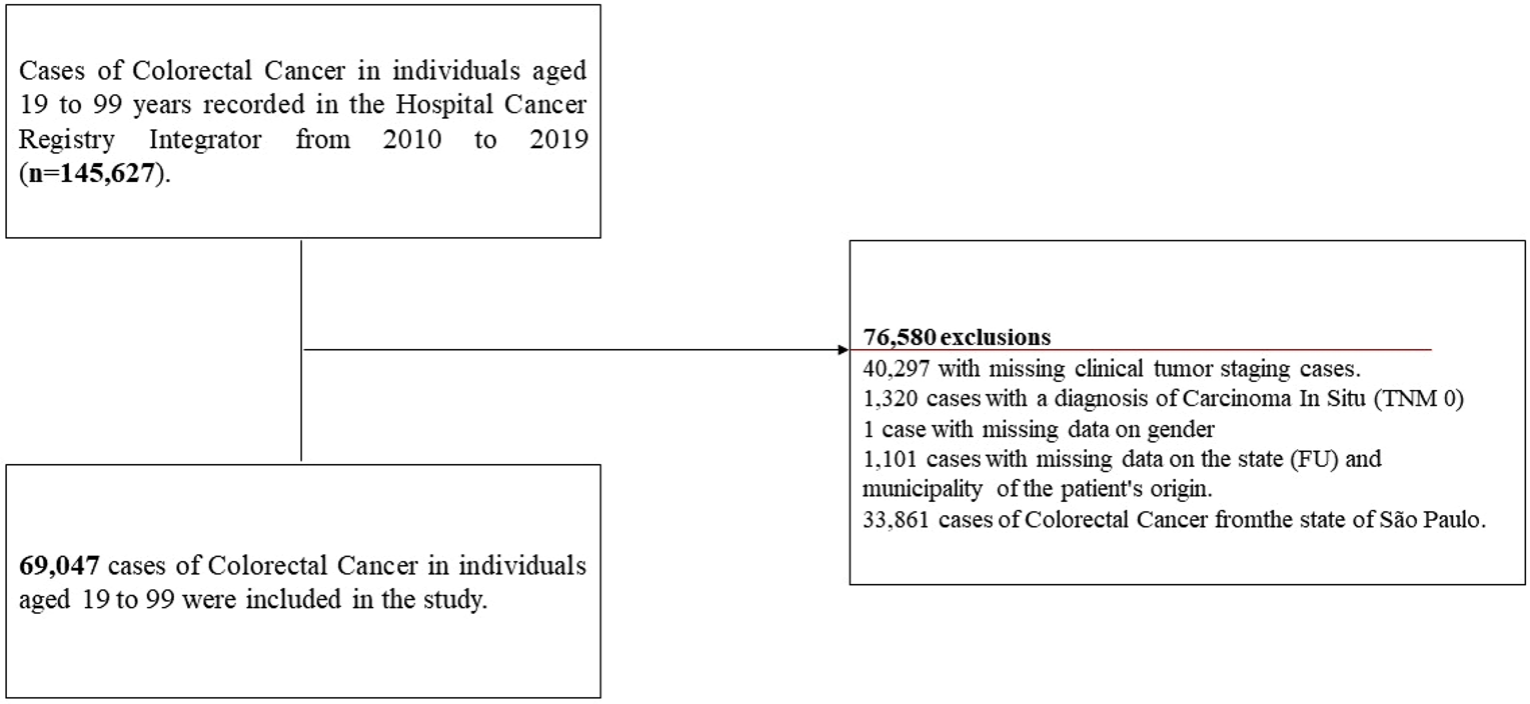
Flowchart of the colorectal cancer case selection process description. Flowchart depicting the description of the selection process of colorectal cancer cases from 2010 to 2019 in the hospital hancer hegistry integrator. *TNM* Classification of Malignant Tumours; *FU* Federation Unit.

According to the study’s eligibility criteria, 69,047 cases of CRC across all UF in the country were included, with 65.6% (CI 65.2–66.0) in advanced stage and 34.4% (CI 33.9-34.7) in early stage, as presented in Fig. 1.

**Figure 2:**
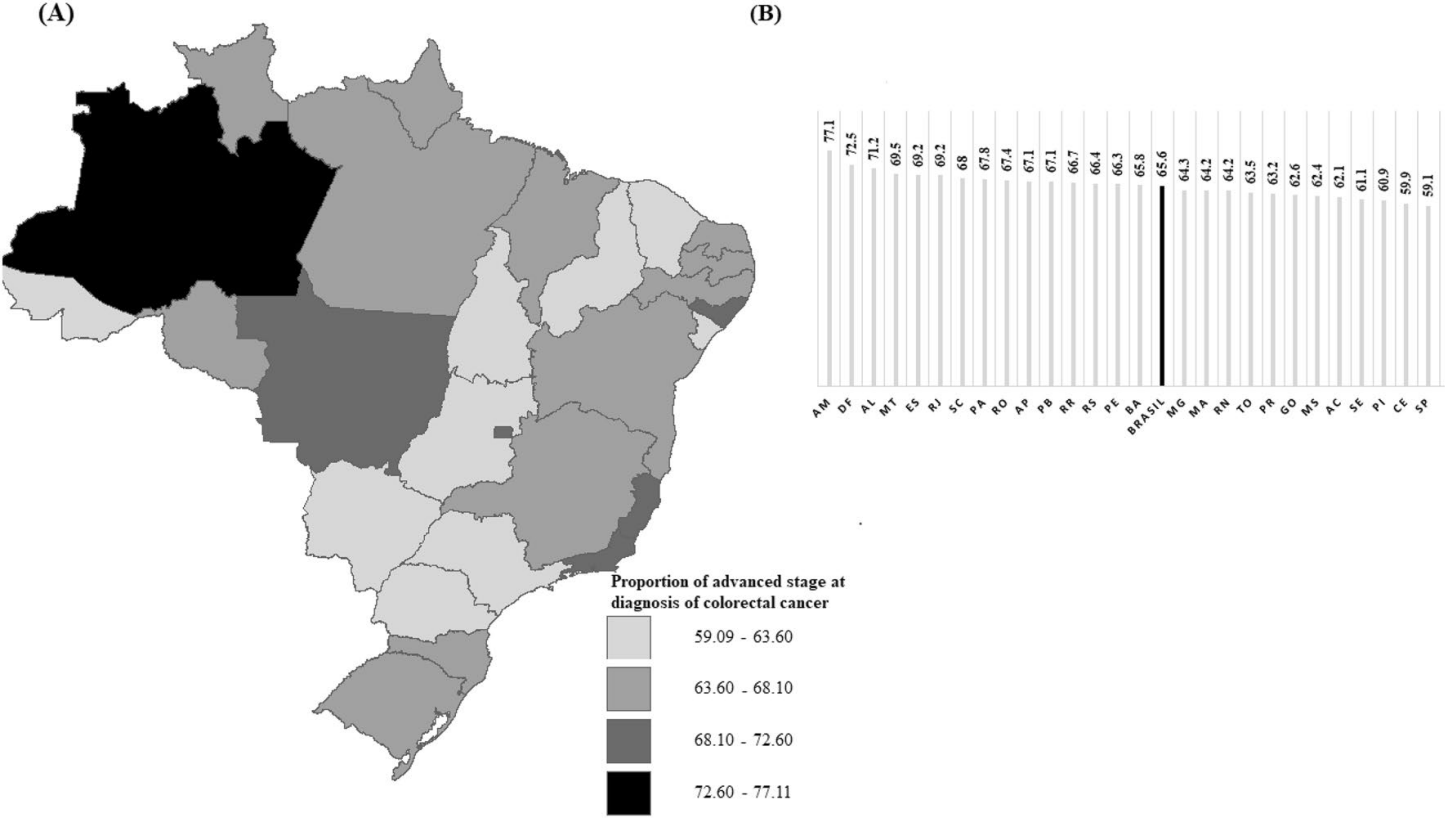
(**A**) Map by UF; (**B**) Graph of the proportions by FU. Spatial distribution of the proportion of advanced-stage diagnosis for colorectal cancer. Spatial distribution of the proportion of advanced stage diagnosis (TNM stage III and IV) for colorectal cancer, from 2010 to 2019, by FU (n = 102,908). Website of the territorial meshes https://www.ibge.gov.br/geociencias/organizacao-do-territorio/malhas-territoriais.html Public domain and not protected by copyright. Software: TerraView 4.2.2 https://www.dpi.inpe.br/terralib5/wiki/doku.php?id=wiki:downloads:terraview_terralib_4.2.2. AM: Amazonas; DF: Distrito Federal; AL: Alagoas; MT: Mato Grosso; ES: Espirito Santo; RJ: Rio de Janeiro; SC: Santa Catarina; PA: Pará; RO: Rondônia; AP: Amapá; PB: Paraíba; RR: Roraima; RS: Rio Grande do Sul; PE: Pernambuco; BA: Bahia; MG: Minas Gerais; MA: Maranhão ; RN: Rio Grande do Norte; TO: Tocantis; PR: Paraná; GO: Goiás; MS: Mato Grosso do Sul; AC: Acre; SE: Sergipe; PI: Piauí; CE: Ceará; SP: São Paulo.

Figure 2 spatially presents the distribution of proportions of advanced stage at diagnosis of CRC for all 26 Brazilian FU and the Federal District. The proportion of advanced stage at diagnoses of CRC in Brazil from, varying among states and regions of the country.

The highest proportions of advanced stage at diagnosis of this condition are located in the North, Central-West, and Northeast regions, with emphasis on Amazonas (77.1%), Distrito Federal (72.5%), and Alagoas (71.2%).

**Figure 3:**
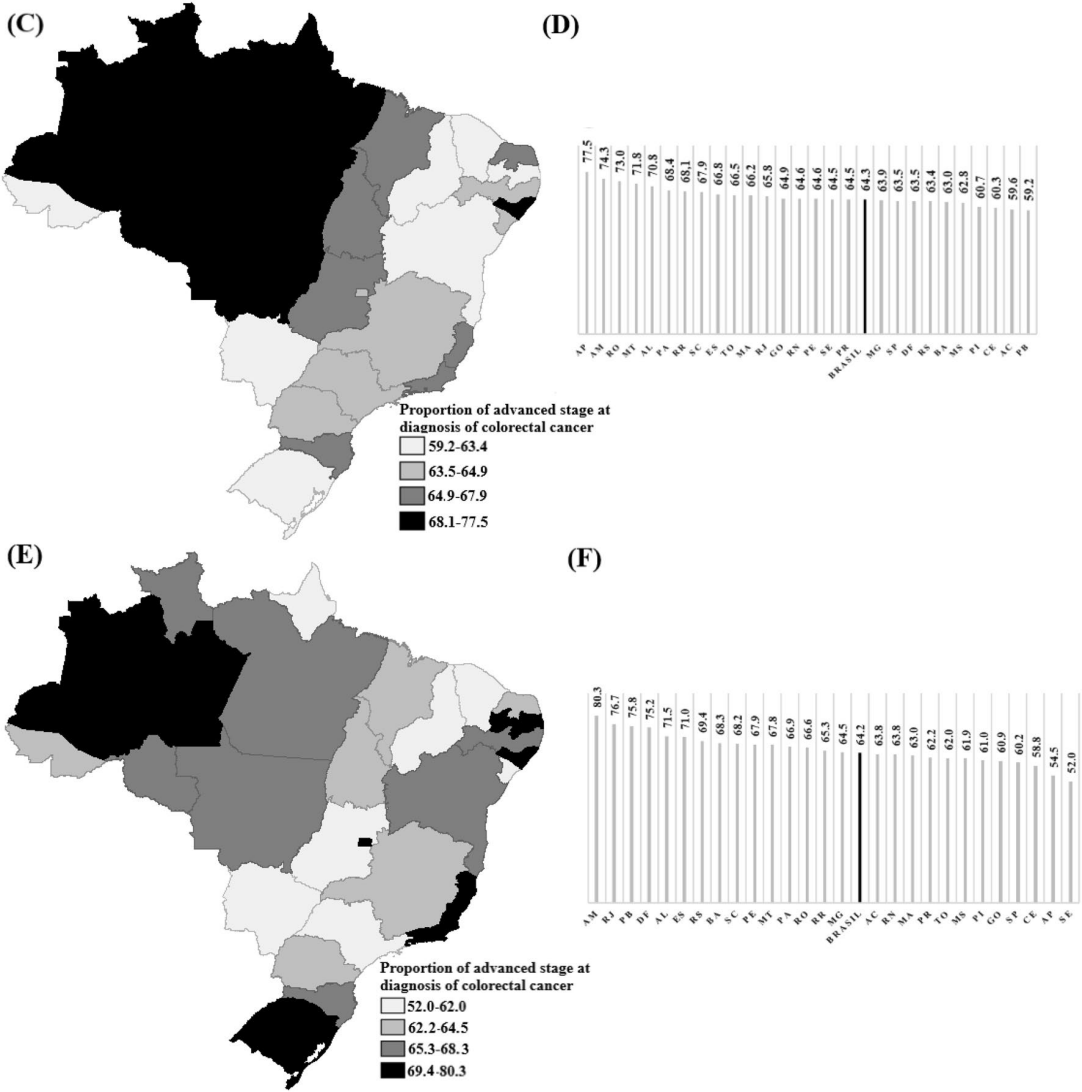
(**C**) Map 2010 to 2014; (**D**) Graph of the proportions by FU of the first period; (**E**) Map 2015 to 2019; (**F**) Graph of the proportions by FU of the second period. Spatial distribution of the comparison between periods of the proportion of advanced stage diagnoses for colorectal cancer. Spatial distribution of the proportion of advanced stage CRC at diagnosis (TNM staging III and IV), with a comparative analysis between the periods 2010 to 2014 and 2015 to 2019, by Brazilian states (n = 102,908). Website of the territorial meshes https://www.ibge.gov.br/geociencias/organizacao-do-territorio/malhas-territoriais.html Public domain and not protected by copyright. Software: TerraView 4.2.2 https://www.dpi.inpe.br/terralib5/wiki/doku.php?id=wiki:downloads:terraview_terralib_4.2.2. AM: Amazonas; DF: Distrito Federal; AL: Alagoas; MT: Mato Grosso; ES: Espirito Santo; RJ: Rio de Janeiro; SC: Santa Catarina; PA: Pará; RO: Rondônia; AP: Amapá; PB: Paraíba; RR: Roraima; RS: Rio Grande do Sul; PE: Pernambuco; BA: Bahia; MG: Minas Gerais; MA: Maranhão ; RN: Rio Grande do Norte; TO: Tocantis; PR: Paraná; GO: Goiás; MS: Mato Grosso do Sul; AC: Acre; SE: Sergipe; PI: Piauí; CE: Ceará; SP: São Paulo.

Figure 3 spatially presents a comparative of the distribution of proportions of advanced stage CRC at diagnosis for all 26 Brazilian states and the Federal District. It is noted that the proportions remain consistent over time, with differences in the distribution pattern among the states with the highest and lowest values.

The majority of diagnosed cases of advanced stage CRC were in men, in the age range of 60 to 69 years, were white, illiterate, or had incomplete primary education, as shown in Table 2. In Supplementary Table S3, other variables that were also analyzed for the investigated outcome were presented.

**Table 2:** Prevalence and unadjusted prevalence ratios for advanced stage colorectal cancer, according to individual characteristics and contextual variables.

Advanced stage of colorectal cancer					
	n	%	PR	CI (95%)	p*
Individual variables					
Sex					
Female	22.186	64.7	1.06	1.04–1.08	< 0.001*
Male	23.142	66.6	1.00	–	
Age group					
18–49 years old	4.802	71.0	1.86	1.77–1.95	
50–59 years old	14.154	83.6	1.05	1.01–1.10	< 0.001*
60–69 years old	19.300	84.2	1.00	–	
70 years or older	7.072	31.4	4.40	4.26–4.54	
Race					
White	23.746	65.0	1.06	1.04–1.08	< 0.001*
Non white	17.012	58.9	1.00	–	
No information	4.570	64.0	1.09	1.05–1.13	
Primary tumor location					
Right	16.499	66.2	1.02	1.00–1.02	< 0.02*
Left	28.379	65.3	1.00	–	
Education					
None/incomplete fundamental education	18.993	62.9	1.36	1.29–1.44	
Fundamental education	7.450	66.8	1.22	1.15–1.29	
Secondary education/incomplete	7.386	71.3	1.05	0.99–1.12	< 0.001*
Undergraduate education	2.761	72.7	1.00	-	
No information	8.738	64.5	1.30	1.23–1.38	
Socioeconomic contextual variables					
Gini index					
0.49–0.56	30.175	65.3	1.00	–	
0.59–0.55	15.153	66.2	1.01	1.00–1.02	< 0.02*
HDI					
0.631–0.731	22.750	64.8	1.28	1.12–1.46	
0.735–0.774	22.164	66.3	1.22	1.07–1.40	< 0.001*
0.783–0.824	414	72.5	1.00	–	
Health service offer contextual variables					
Density of family health strategy teams (per 100,000 inhabitants)					
0.37–5.75	13.087	64.3	1.04	1.02–1.08	< 0.001*
5.86–8.53	12.028	66.9	0.97	0.94–0.99	
8.62–13.26	20.213	65.7	1.00	–	
Density of oncologist (per 100,000 inhabitants)					
5.72–28.24	22.143	65.3	1.00	0.98–1.02	
29.56–40.92	17.472	66.3	1.02	1.00–1.04	< 0.01*
41.23–60.10	5.173	65.1	1.00	–	
Density of coloproctologist (per 1000,000 inhabitants)					
0.00–1.06	13.567	64.7	0.99	0.98–1.01	
1.40–1.87	15.030	64.6	1.04	1.03–1.05	< 0.001*
1.99–10.41	16.731	67.4	1.00		
Density of gastroenterologist (per 100,000 inhabitants)					
3.15–15.84	21.858	64.6	1.17	1.13–1.22	
16.12–26.83	18.786	65.8	1.14	1.09–1.18	< 0.001*
30.08–36.60	4.684	69.9	1.00	-	
Density of oncology services (per 100,000 inhabitants)					
1.31–5.34	21.935	64.9	1.07	1.05–1.10	< 0.001*
5.26–7.72	8.423	64.8	1.08	1.04–1.11	
7.81–14.34	14.970	67.3	1.00	-	
Colonoscopy exams (per 100,000 inhabitants)					
0.21–112.73	23.969	65.6	1.11	1.08–1.15	< 0.001*
117.35–239.14	15.887	64.6	1.17	1.10–1.19	
273.88–388.35	5.472	69.1	1.00	–	

Brazil, by place residence, excluding SP (n = 69,047).

*PR* estimated prevalence ratio by robust poisson model, *CI* confidence interval; *p* wald’s test, *SP* São Paulo.

*Statistically significant.

For the bivariate and multilevel analysis of the data, it was decided to exclude all CRC cases from the Cancer Hospital Registry (RHC) of the state of São Paulo (n = 33,861), as individual socio-economic information of the cancer cases was not available, which would not allow for data comparability. A total of 69,047 CRC cases were included in these analyses.

The unadjusted prevalences and PR for advanced stage CRC are presented in Table 2. Statistically significant associations are observed between advanced stage CRC and all individual variables, as well as contextual socioeconomic and healthcare service variables.

Table 3 presents the results regarding the multilevel analysis of the data. The multilevel modeling, through the initial empty model, provides evidence that the variation across Brazilian FU is statistically different from zero, according to the likelihood ratio test for CRC (LR 58.54; p < 0.001).

**Table 3:** Multilevel analysis among individual and contextual variables for advanced colorectal cancer stage in individuals aged 18 to 99 years old in the period from 2010 to 2019.

Variables	Empty model	Model 1		Model 2	
		PR (IC 95%)	p	PR (CI 95%)	p
Level 1 (Individual)					
Age group					
18—49 years old	**–**	11.84 (1.74–1.94)		1.84 (1.74–1.94)	
50—59 years old	**–**	1.05 (0.99–1.10)	0.001*	1.05 (0.99–1.10)	0.001*
60—69 years old		1		1	
70 years or older	**–**	4.40 (4.25–4.57)		4.40 (4.25–4.57)	
Education					
None/incomplete fundamental education	**–**	1.02 (0.95–1.08)		1.02 (0.95–1.08)	
Fundamental education	**–**	1.03 (0.96–1.10)	0.24	1.03 (0.96–1.10)	0.25
Secondary education/incomplete	**–**	1.06 (0.98–1.13)		1.06 (0.98–1.13)	
Undergraduate education	**–**	1		1	
No information	**–**	1.01 (0.95–1.09)		1.01 (0.95–1.09)	
Level 2 (Aggregated by FU)					
HDI					
0.631–0.731	**–**	**–**	**–**	1.22 (1.01–1.49)	
0.735–0.774	**–**	**–**	**–**	1.19 (0.97–1.45)	0.04*
0.783–0.824	**–**	**–**	**–**	1	
Density of family health strategy teams					
0.37–5.75	**–**	**–**	**–**	1.10 (1.03–1.21)	
5.86–8.53	**–**	**–**	**–**	1.07 (1.02–1.16)	0.02*
8.62–13.26	**–**	**–**	**–**	1	
Fixed effects					
Intercept (CI 95%)	− 1.084 (− 1.13–− 1.04)	0.152 (0.14–0.16)		0.127 (0.10–0.16)	
Random effects					
Variance (CI 95%)	0.0912 (0.061–0.137)	0.0045 (0.001–0.011)		0.0033 (0.001–0.009)	
LR Test (x^2^. p-value)	58.54 (< 0.001)	42.98 (< 0.001)		29.14 (< 0.001)	

Brazil, according to place residence, except SP (n = 69,047).

Model 1: Statistical model with inclusion of individual level variables; Model 2: Statistical model with inclusion of variables of individual level and contextual level per FU.

*PR* prevalence ratio adjusted by the multilevel model with random intercept, *CI *confidence interval, *p* wald’s test, *SP*: São Paulo.

*p trend ≤ 0.05.

The results of the multilevel analysis for CRC indicate that the advanced stage CRC at diagnosis is associated with older age groups (PR 4.40; CI 4.25–4.57), as well as younger age groups (PR 1.84; CI: 1.74–1.94), compared to the age group of 60 to 69 years. The advanced stage CRC at diagnosis was also significantly associated with lower HDI (PR 1.22; CI 1.01–1.49) and lower density of the family health strategy team (PR 1.10; CI 1.03–1.21), as shown in Table 3.

## Discussion

Both contextual factors and healthcare service availability were significant determinants of advanced stage CRC at diagnosis. The prevalence of advanced stage CRC at diagnosis was 65.6% (CI 65.2—66.0), with significant variations among the FUs.

This high proportion of advanced stage CRC at diagnosis is a reality observed in several places around the world. A study conducted in Sub-Saharan Africa identified that 66% of individuals with known stages were diagnosed at a late stage^16^, similar findings were observed in Malaysia^17^, Northwest Amhara, Ethiopia^18^, the United Kingdom^19^, and the United States^20^.

Possible associated factors may include the absence of early diagnosis or screening programs, a lack of adequately trained professionals, and the stigma associated with cancer, which can interfere with the utilization of diagnostic investigations in certain communities^21^.

Advanced stage CRC at diagnosis when compared among the Brazilian UF in this research, based on the theoretical model of SDH, was associated at the level of intermediate determinants with both older and younger age groups (biological factor), within the sociopolitical context, with low Human Development Index (HDI) (socioeconomic contextual conditions), and also at the level of intermediate determinants with lower density of family health strategy teams (local contextual indicators related to the provision and access to healthcare services).

CRC primarily affects men and women over the age of 50^22^. Older individuals have a much higher risk of CRC than those under 50 years old^23^. Adenomas are present in up to 20% of individuals over the age of 50, and in many cases, up to 50%, with the majority of CRC developing from adenomas through the adenoma-carcinoma pathway^24^.

In this study, individuals aged 70 years or older had a prevalence four times higher of being diagnosed at an advanced stage compared to younger age groups. This finding is consistent with a study conducted in Korea where elderly patients had a higher prevalence of right-sided cancer with obstruction and advanced stage^25^. This finding can be explained by the fact that the aging process is associated with a progressive depletion of functional reserve in multiple organs and systems, increased prevalence of comorbidities, and decreased social and economic resources at a time when they are most needed for healthcare-related costs^26^, which can lead to a delay in diagnosis.

It is important to emphasize that in our study, younger age groups with CRC also had a higher prevalence of diagnosis at an advanced stage. Siegel et al. demonstrated in their research that the incidence of early-onset CRC has been increasing over the past three decades in the United States. The incidence rates of CRC show a rising trend of nearly 45% in adults aged 20 to 49, from 8.6 per 100,000 in 1992 to 13.1 per 100,000 in 2016 in the United States at a more advanced stage^24^. The same trend was observed in New Zealand at 4.0%, Canada at 2.8%, and Australia at 2.2%^27^.

These patients generally have lower awareness of CRC, are not part of screening programs, tend to underestimate symptoms, and are reluctant to seek medical care, which can contribute to delays in diagnosis and consequently present an advanced stage of the disease^28^. Cases in young individuals can also arise from high-risk hereditary populations such as Lynch Syndrome, Familial Adenomatous Polyposis, and Juvenile Polyposis Syndrome^29^.

Such a result may also be a consequence of the discharacterization of social health policies that Brazil has been experiencing since 2015, especially in primary care, with a reduction in the team that makes up this level of attention. This hinders access to early diagnosis and referral to other levels of care in the system that can provide specialized care^30^.

Beyond issues related to the context of intermediate determinants concerning the individual in the development of CRC, it is essential to conduct a broader analysis that incorporates the social determinants of health at the sociopolitical level in this regard, which, according to the WHO, are “the circumstances in which people are born, grow, live, work, and age, and the wider set of forces and systems shaping the conditions of daily life”^31^. It is necessary to address these determinants in order to achieve more effective control over cancer^32^.

Low HDI was associated with advanced staging in this study. HDI is a composite measure of a country’s average life expectancy, years of education, and per capita income^33^, serving as a parameter that reflects the social inequalities within a territory, which has implications for disparities in overall CRC care^34^. This relation may be explained by the impact of social disparities on the continuum of CRC care.

The literature also indicates that even when diagnosed at an early stage, the risk of death among CRC patients is twice as high in countries with low HDI compared to those with high HDI, suggesting that factors associated with HDI play an important role in prognosis regardless of the stage^35^. Low education levels can influence a lack of awareness regarding early signs and symptoms, and low per capita income may be associated with limited access to healthcare services for screening, diagnostic confirmation, and treatment purposes^36^.

Individuals with CRC in this study had a higher prevalence of advanced staging in areas where there is a low density of family health strategy teams, indicating a deficiency in the healthcare workforce dedicated to their care. In Brazil, according to the National Policy for Cancer Prevention and Control (PNPCC), early cancer detection should primarily be carried out within the scope of Primary Care^37^ which has the basic health unit and the family health strategy team as structuring axes, which, according to the organization of the healthcare network, should refer patients with suspected signs and symptoms of colon and rectal cancers to specialized care in order to investigate and confirm the diagnosis^38^. Primary care is usually where CRC screening takes place, particularly when it involves fecal occult blood testing.

Favorable results related to CRC diagnosis were associated with visits to primary care physicians (PCPs) as they serve as the first point of contact for patients within the healthcare system^39^, responsible for discussing and recommending screening and referring patients with suspected signs and symptoms to specialists. As a result, there is a lower incidence of advanced-stage CRC and higher survival rates when such professionals are available^40,41^.

Reducing clinical staging has been identified as a more cost-effective solution among the actions to control the cancer burden. This strategy involves increasing public awareness of early signs and symptoms, educating frontline healthcare professionals, and improving referral procedures to enable immediate and accurate diagnosis, as well as treatment of cancer in its early stages^42^.

Screening programs help reduce the impact of CRC on the population through early detection and even by reducing the incidence of CRC through the identification of adenomatous polyps^43^. In Brazil, for the Unified Health System (SUS), prioritizing actions for early diagnosis of symptomatic cases and providing personalized approaches for high-risk situations is recommended, given the lack of capacity to guarantee the entire continuum of care for screening. Screening is recommended for individuals aged 50 to 75 in contexts where continuity of care can be ensured^44^. There is actually no effectively established CRC screening program in the country, with only a few local pilot projects^45^.

The results of the present research point to inequities in access to specific health services and care provided in Brazil. These high proportions of advanced stage at diagnosis for CRC in the country highlight the deficit in the capacity to promote early detection, diagnosis, and treatment, which directly impacts the survival of those living with these conditions.

Regions with greater social, cultural, and economic inequalities experience longer delays in accessing healthcare services, diagnosis, and initiation of treatment, which will impact cancer staging and, consequently, reduce the effectiveness of treatment, quality of life, and survival for those living with this disease^18,35,40^.

Social inequities impact all aspects of cancer, from research to healthcare systems, from disparities in incidence to treatment outcomes, and in the lives of individuals after this disease^46^. They are already identified as strong predictors of morbidity and premature mortality worldwide and contribute to cancer inequalities within and between countries^21^. This is a reality identified in Brazil, where projections for premature mortality rates due to CRC in the period of (2026–2030) demonstrate an expected increase in both men and women in all regions, except in the Southeast (where rates are expected to remain stable) as it is one of the wealthiest regions in the country^47^.

Therefore, in order to improve early detection and prevention of CRC across all age groups in the country, it is necessary to create control plans for these conditions that seek to use educational measures at both the individual and community levels, develop screening strategies, provide training, and increase the workforce in digestive endoscopy. It is important to address and reduce disparities in the concentration of specialized services and customize screening and early diagnosis strategies to fit local resources and realities, in order to ensure equitable access to CRC care^48,49^.

It is important to emphasize that the results of this study also indicate that the pattern of missing data for the clinical staging of the primary tumor is associated with individual and contextual factors in cases of colorectal malignant neoplasms. Younger individuals present a higher prevalence for missing staging data, as well as the limited availability of specialized medical professionals in oncology and proctology, and low density to specialized oncology services are determining factors in the pattern of data loss, highlighting the need for improvement in cancer information in the country.

Limitations of this study include the use of secondary data sources from the Brazilian health information systems. However, the RHC is the most comprehensive secondary data source available in the country regarding cancer diagnosis^50^, due to its multiplicity of primary approaches, colorectal cancer can influence data recording with duplicates. Furthermore, prior measures for control and adjustment of variables were employed, ensuring the reliability of the presented results. Another limitation is the cross-sectional design of the study, which does not allow for establishing a temporal relationship between the investigated outcome and the analyzed factors.

Although we had a large number of cases in our study, few associations were observed, which was unexpected and may indicate how these factors operate differently depending on the population being analyzed. It is pertinent to emphasize that caution should be exercised in interpreting a p-value, as this measure is heavily influenced by sample size. Large samples tend to produce small p-values, even if the observed effect is not of great practical importance, while small samples tend to produce large p-values, even if there is an important practical effect.

The information presented in this study allows us to conclude that the advanced stage CRC at diagnosis of in Brazil is associated with limited access to healthcare services, as well as the unequal distribution of social determinants at the intermediate level and within the sociopolitical context in the country.

The presented results demonstrate the impact that individual and contextual social determinants have on individuals diagnosed with advanced stage CRC in the country. The adoption of broader actions is necessary to improve the HDI of states through social public policies to reduce existing inequities. In the health field, improvements in disease management are needed, with accessibility to healthcare facilities, primary prevention strategies through health promotion actions for a healthy lifestyle, early detection through the implementation of a screening program, and organized medical assistance to promote timely investigation that can identify cancer at an early stage, improving the prognosis for those living with such conditions in Brazil.

## Methods

### Study design and participants

This is an observational cross-sectional study conducted using secondary data collected from the Hospital Cancer Registry (RHC). The RHC provides standardized information on sociodemographic aspects of cancer patients, clinical characteristics of tumors, and hospital care activities^51^.

The RHC are expanding throughout the country, covering over 80% of the high-complexity oncology network units in the SUS across the 26 Brazilian states and the Federal District. Data access is provided through the sisRHC developed by INCA and made available through open access via the Integrador RHC web system. This system allows quick and easy access to a nationwide database, serving as a crucial tool for analyzing the quality of oncological care in Brazil. In these hospitals, patients referred by the public health services network are treated^51^.

Cases of malignant neoplasms of colon (C18) and rectum (C19/C20) were analyzed, selected according to the International Classification of Diseases 10th revision (ICD-10/ICD-3)^52^, in individuals of both sexes aged between 18 and 99 years, treated in oncology care units, diagnosed from 2010 to 2019.

Cases with missing data on TNM staging of the tumor, carcinoma in situ (TNM 0), and absence of information regarding age and place of residence at the time of diagnosis were excluded from this study. Only new cases diagnosed within the established period were included in the study.

Data from the HCR of the state of São Paulo were not included in the analysis due to its different data collection processes, with no individual information available on the socioeconomic conditions of cancer patients. Including this HCR would prevent data comparison.

### Variables

The outcome analyzed in this research is advanced tumor clinical stage (III and IV), classified by the TNM System for Classification of Malignant Tumors. This system is based on the anatomical extent of the tumor, analyzing the characteristics of the primary tumor, the proximal lymph nodes, and the presence or absence of distant metastasis^53^. The clinical staging of the primary tumor was dichotomized into advanced stage (TNM III and IV) and early stage (TNM I and II).

The independent variables (Fig. 3) were grouped based on the theoretical conceptual model of the Social Determinants of Health (SDH) developed by the Commission on Social Determinants of Health (CSDH) of the World Health Organization (WHO). This conceptual model includes three dimensions of SDH: Sociopolitical context; Socioeconomic position; and Intermediate determinants of health^54^.

**Figure 4:**
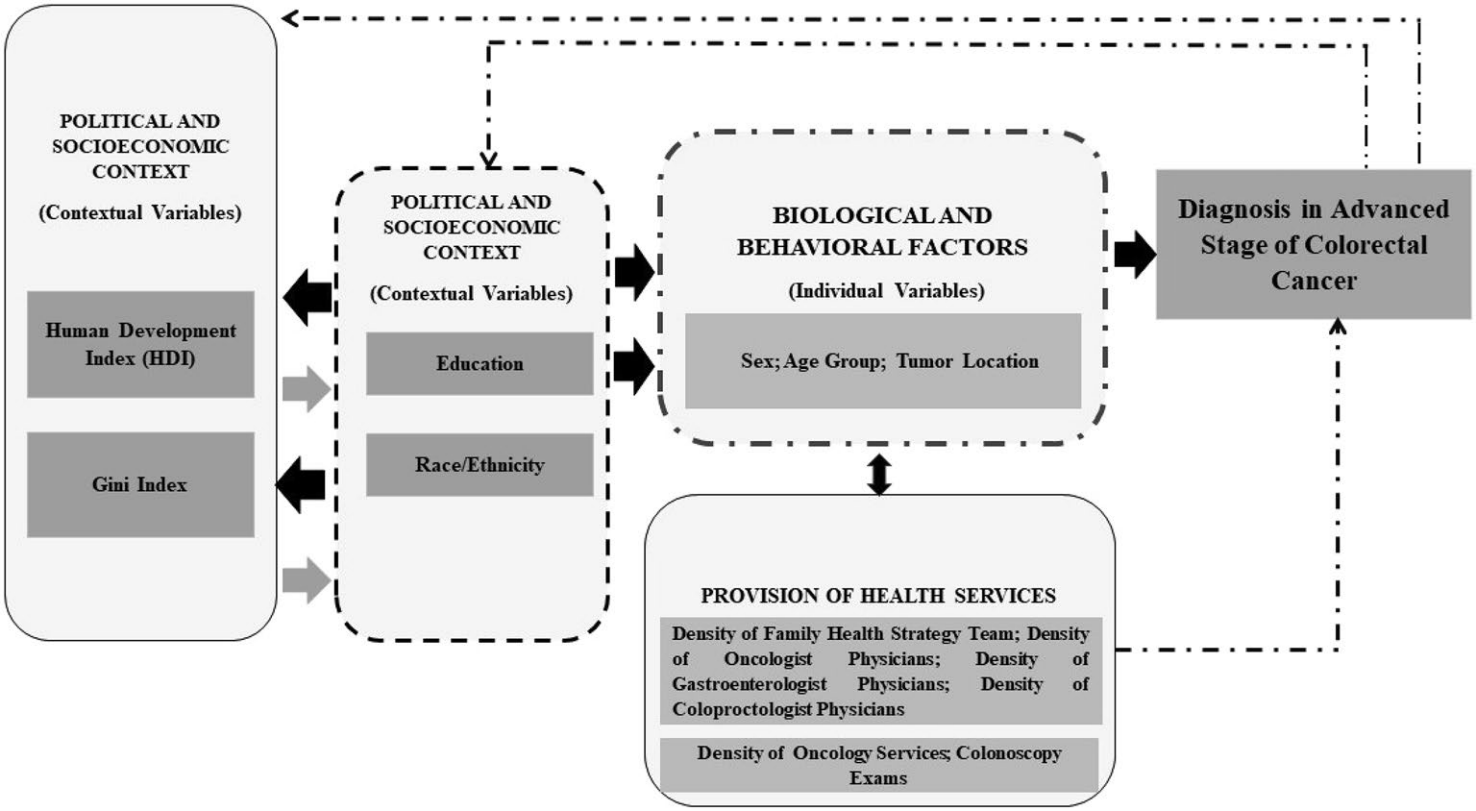
Theoretical explanatory model of Social Determinants of Health linked to the diagnosis of advanced stage colorectal cancer in Brazil.

In the sociopolitical context, variables related to the socioeconomic contextual conditions of Brazilian states (Human Development Index—HDI) were included. Under the structural determinants, individual variables related to the socioeconomic position of individuals diagnosed with colorectal cancer (education level) were grouped. In the intermediate determinants, biological and behavioral factors were combined with local contextual indicators related to the provision and access to healthcare services (age group and density of family health strategy teams). Figure 4 presents the variables of the study according to the indicated theoretical model.

### Data sources

The individual databases were associated with two other databases with aggregated data per Federation Unit (FU): (a) data collected from the Atlas of Human Development in Brazil, made available by the United Nations Development Programme (UNDP)^55^, and (b) data on medical density and offer of health services, extracted from the National Registry of Health Institutions (NRHI)^56^, the Outpatient Health Information System (SIA)^57^. From these data, specific indicators were calculated for the years 2012 and 2017: “ Density of Family Health Strategy Teams “ (Number of teams composed of a Nurse, Dentist, and Physician per 100,000 inhabitants), “Density of Family Doctor” ( Number of Medical Family per 100,000 inhabitants) “ Density of Oncologist” (Number of Medical Oncologists per 100,000 inhabitants), “ Density of Coloproctologist” (Number of Coloproctologists per 10000,000 inhabitants), “ Density of Gastroenterologist “ (Number of Gastroenterologists per 100,000 inhabitants), “ Density of Oncology Services” (Number of Accredited Oncology Services per 100,000 inhabitants), “Health Insurance Plan Density” (Number of number of insured individuals with health insurance per 100,000 inhabitants**),** “ Colonoscopy Exams” (Number of colonoscopy exams per 100,000 inhabitants), “Rectosigmoidoscopy Exams” (Number of Rectosigmoidoscopy per 100,000 inhabitants).

The Brazilian demographic census of 2010 was employed for the calculation of indicators, and population data and estimations per FU, sex, and age were carried out and reported by the Brazilian Institute of Geography and Statistics (IBGE)^58^. Quantitative variables were categorized in tertiles or as dichotomized variables (categorization by the median), when required by bivariate and multilevel data assessment.

### Statistical assessment

The first step was the descriptive analysis of data with a summary of measurements, tabulation, and the construction of graphics.

The maps were elaborated from territorial geographic mesh (shape fles), publicly available from the IBGE, not being protected by copyright. The geographic meshes are made available in: https://www.ibge.gov.br/geociencias/organizacao-do-territorio/ malhas-territoriais.html.

Spatial data analysis was carried out by georeferencing, with software TerraView 4.2.2, utilizing the FU to create specific maps. This analysis shows the spatial distribution of advanced stage CRC at diagnosis across the Brazilian territory between 2010 and 2019.

Pearson’s chi-squared test was applied to verify the association of the dependent variable with the independent variables of the study. Since the analyzed outcome has a prevalence greater than 10% and contextual variables were used, the strategy employed for multivariate analysis was the Poisson Regression model with robust variance. Subsequently, a Multilevel Poisson Regression model was performed, with a random intercept determined according to the results of the Likelihood Ratio (LR) test.

Firstly, an empty model was analyzed, only with random intercept. Individual level variables were included, with random intercept—the reduction in the variability of the random effect was examined by comparing with the previous model. Then, contextual level variables were included in the modeling. The statistically significant variables were maintained in the model, according to Wald’s test (α = 0.05), along with those variables that presented theoretical plausibility for being included in the final statistical model. The individual interactions between the first and second-level variables were also tested separately, with no interaction found between them. Subsequently, a new variable was created by combining a first-level socioeconomic variable with a second-level variable. Then, a new model was run to observe the behavior of the variance and the model as a whole. The result showed that the interaction term was not significant and did not alter the variance of the previous model (0.003) for advanced staging and (0.02) for missing staging, indicating an absence of “cross-level interaction”^59^.

The p trend test was carried out to determine the dose–response effect between the independent variables of the study and the prevalence of advanced stage CRC at diagnosis, including the variables that constitute the final multilevel model^59^. All analyses were developed with Software Stata 15.1.

### Ethical declarations

As this study was conducted using aggregated data from secondary sources, extracted from official websites open to public consultation, and it is not possible to identify individuals, the data used in this research comply with the guidelines and regulations of the Research Ethics Committee (CEP), which exempts registration in the system, in accordance with Resolution 580/2018 used in the country^60^.

### Supplementary Information

Supplementary Tables.

## Data Availability

The primary aggregated data used in this study were extracted from official websites open for public consultation, which are available at the following links: https://www.ibge.gov.br/, https://irhc.inca.gov.br/RHCNet/, http://www.atlasbrasil.org.br/, https://datasus.saude.gov.br/cnes-equipes-de-saude, https://datasus.saude.gov.br/cnes-recursos-humanos-a-partir-de-agosto-de-2007-ocupacoes-classificadas-pela-cbo-2002, https://datasus.saude.gov.br/acesso-a-informacao/producao-ambulatorial-sia-sus/. The database that was organized from the primary data to support the results of this study is available from the corresponding author upon request.
