# Author Correction: Multilevel analysis of social determinants of advanced stage colorectal cancer diagnosis

**DOI:** 10.1038/s41598-024-66240-5

**Published:** 2024-07-08

**Authors:** Amanda Almeida Gomes Dantas, Nayara Priscila Dantas de Oliveira, Guilherme Augusto Barcello Costa, Luís Felipe Leite Martins, Jonas Eduardo Monteiro dos Santos, Arn Migowski, Marianna de Camargo Cancela, Dyego Leandro Bezerra de Souza

**Affiliations:** 1 Graduate Program in Health Sciences, Federal University of Rio Grande do Norte – UFRN, Natal, RN Brazil; 2 Department of Physical Therapy, University of Pernambuco – UPE, Petrolina, PE Brazil; 3 Graduate Program in Oncology, Research and Innovation Coordination, National Cancer Institute (INCA), Ministry of Health, Rio de Janeiro, RJ Brazil; 4 Surveillance and Situation Analysis Division, Prevention and Surveillance Coordination (CONPREV), National Cancer Institute (INCA), Ministry of Health, Rio de Janeiro, RJ Brazil; 5 Division of Clinical Research and Technological Development, Research and Innovation Coordination, National Cancer Institute (INCA), Ministry of Health, Rio de Janeiro, RJ Brazil; 6 Professional Master’s Program in Health Technology Assessment, Education and Research Coordination, National Institute of Cardiology (INC), Ministry of Health, Rio de Janeiro, RJ Brazil; 7 Graduate Program in Public Health, Federal University of Rio Grande do Norte – UFRN, Natal, RN Brazil; 8 Methodology, Methods, Models and Results in Health and Social Sciences Research Group (M3O), Faculty of Health Sciences and Well-Being. Health and Social Care Research Center (CESS), University of Vic-Central University of Catalonia (UVic-UCC), Vic, Spain; 9 Public Health Department, Graduate Program in Public Health, Federal University of Rio Grande do Norte, 1787 Senador Salgado Filho Ave., Lagoa Nova, Natal, RN 59010-000 Brazil

Correction to: *Scientific Reports* 10.1038/s41598-024-60449-0, published online 26 April 2024

The original version of this Article contained errors in Affiliation 5 and Affiliation 6, which were incorrectly given as ‘Epidemiology Unit. Education and Research Coordination, National Institute of Cardiology (INCA), Ministry of Health, Rio de Janeiro, RJ, Brazil.’ and ‘Prevention and Surveillance Coordination (CONPREV), National Cancer Institute (INCA), Ministry of Health, Rio de Janeiro, RJ, Brazil.’, respectively. The correct affiliations are listed below:

5 Division of Clinical Research and Technological Development, Research and Innovation Coordination, National Cancer Institute (INCA), Ministry of Health. Rio de Janeiro-RJ, Brazil.

6 Professional Master’s Program in Health Technology Assessment, Education and Research Coordination, National Institute of Cardiology (INC), Ministry of Health. Rio de Janeiro-RJ, Brazil.

The original Article and accompanying Supplementary Information file have been corrected.

