# Supplementary material for: Multilevel analysis of social determinants of advanced stage colorectal cancer diagnosis
Source: Sci Rep. 2024 Apr 26;14:9667. doi: 10.1038/s41598-024-60449-0 (PMC11053035; doi:10.1038/s41598-024-60449-0)
Supplement: Supplementary file 1 — Supplementary Tables. [file 41598_2024_60449_MOESM1_ESM.pdf]

MULTILEVEL ANALYSIS OF SOCIAL DETERMINANTS OF ADVANCED STAGE COLORECTAL CANCER DIAGNOSIS

Amanda Almeida Gomes Dantas[1]; Nayara Priscila Dantas de Oliveira[2]; Guilherme Augusto Barcello Costa[3];Luís Felipe Leite Martins[4]; Jonas Eduardo Monteiro dos Santos[4]; Arn Migowski [5,6]; Marianna de Camargo Cancela[3]; Dyego Leandro Bezerra de Souza [7,8*].

[1] Graduate Program in Health Sciences, Federal University of Rio Grande do Norte – UFRN. Natal-RN, Brazil. e-mail: almmeidaamanda@gmail.com

[2] Department of Physical Therapy, University of Pernambuco– UPE. Petrolina-PE, Brazil. e-mail: nayara.oliveira@upe.br

[3] Graduate Program in Oncology, Research and Innovation Coordination; National Cancer Institute (INCA), Ministry of Health. Rio de Janeiro-RJ, Brazil. e-mail: guilherme.costa@inca.gov.br; email:marianna.cancela@inca.gov.br

[4] Surveillance and Situation Analysis Division, Prevention and Surveillance Coordination (CONPREV); National Cancer Institute (INCA), Ministry of Health. Rio de Janeiro-RJ, Brazil. e-mail: lfmartins@inca.gov.br; e-mail: eduardo.monterio@inca.gov.br

[5]Division of Clinical Research and Technological Development. Research and Innovation Coordination, National Cancer Institute (INCA), Ministry of Health. Rio de Janeiro-RJ, Brazil. e-mail: arn.santos@inca.gov.br

[6]Professional Master's Program in Health Technology Assessment. Education and Research Coordination. National Institute of Cardiology (INC), Ministry of Health. Rio de Janeiro-RJ, Brazil. e-mail: arn.santos@inca.gov.br

[7] Graduate Program in Public Health, Federal University of Rio Grande do Norte – UFRN. Natal-RN, Brazil. e-mail: dysouz@yahoo.com.br

[8] Methodology, Methods, Models and Results in Health and Social Sciences Research Group (M3O), Faculty of Health Sciences and Well-being. Health and Social Care Research Center (CESS); University of Vic-Central University of Catalonia (UVic-UCC), Vic, Spain. e-mail: dysouz@yahoo.com.br

	Missing Stage Of Colorectal Cancer				
	n	%	PR	CI (95%)	p*
**Individual Variables**					
**Sex**					
Female	17.787	34.1	1.04	1.03-1.06	<0.001*
Male	16.803	32.6	1.00	-	
**Age Group**					
18 - 49 years old	13.220	66.2	2.94	2.88-3.01	
50 - 59 years old	7.647	31.1	1.38	1.35-1.42	<0.001*
60 - 69 years old	6.615	22.4	1.00	-	
70 years or older	7.108	24.0	1.07	1.04-1.10	
**Race**					
White	14.582	36.4	1.00	-	< 0.001*
Non white	15.837	30.2	1.03	1.02-1.05	
No Information	4.171	36.8	1.21	1.18-1.25	
**Education**					
None/Incomplete fundamental education	11.692	27.9	1.12	1.10-1.14	
Fundamental education	4.977	30.9	1.07	1.05-1.10	
Secondary education/ Incomplete	6.202	37.4	0.97	0.89-0.93	<0.001*
Undergraduate education	2.118	35.8	1.00	-	
No Information	9.601	41.5	0.91	1.11-1.20	
**Socioeconomic contextual variables**					
**Gini Index**					
0.49-0.56	19.991	30.1	1.00	-	
0.59-0.55	14.674	39.0	1.29	1.27-1.32	<0.001*
**HDI**					
0.631-0.731	17.525	33.3	1.34	1.26-1.42	
0.735-0.774	16.483	33.0	1.35	1.27-1.43	<0.001*
0.783-0.824	582	50.5	1.00	-	
**Health service offer contextual variables**					
**Density of Family Health Strategy Teams (per 100,000 inhabitants)**					
0.37-5.75	7.772	27.6	1.11	1.10-1.12	<0.001*
5.86-8.53	10.088	35.9	0.98	0.97-1.00	
8.62-13.26	16.730	35.2	1.00	-	
**Density of Oncologist (per 100,000 inhabitants)**					
5.72-28.24	16.250	32.3	1.02	1.00-1.03	
29.56-40.92	13.881	34.5	0.98	0.99-1.05	<0.01*
41.23-60.10	4.459	33.6	1.00	-	
**Density of Coloproctologist (per 1000,000 inhabitants)**					
0.00-1.06	9.029	30.1	1.12	1.11-1.13	
1.40-1.87	10.441	30.9	1.11	1.10-1.12	<0.001*
1.99-10.41	15.150	37.8	1.00		
**Density of Gastroenterologist (per 100,000 inhabitants)**					
3.15-15.84	16.901	33.3	1.19	1.17-1.21	
16.12-26.83	12.423	30.3	1.24	1.22-1.26	<0.001*
30.08-36.60	5.266	44.0	1.00	-	
**Density of Oncology Services (per 100,000 inhabitants)**					
1.31-5.34	16.805	33.2	1.05	1.04-1.06	<0.001*
5.26-7.72	4.952	27.5	1.14	1.12-1.15	
7.81-14.34	12.833	36.5	1.00	-	
**Colonoscopy Exams (per 100,000 inhabitants)**					
0.21-112.73	20.669	36.1	0.98	0.96-1.00	<0.001*
117.35-239.14	9.619	28.1	1.10	1.09-1.12	
273.88-388.35	4.302	35.2	1.00	-	

PR: Estimated Prevalence Ratio by Robust Poisson Model; CI: Confidence interval; p: Wald's test; *Statistically significant; SP: São Paulo.

Table S1 - Prevalence and unadjusted prevalence ratios for missing stage colorectal cancer, according to individual characteristics and contextual variables. Brazil, by place residence, excluding SP (n = 103, 367).

Variables	Empty model	Model 1		Model 2	
		PR (IC 95%)	p	PR (CI 95%)	p
**Level 1 (Individual)**					
**Age Group**					
18 - 49 years old	_____	2.92 (2.84-3.01)		2.96 (2.88-3.05)	
50 - 59 years old	_____	1.38 (1.33-1.43)	0.001*	1.39 (1.34-1.43)	0.001*
60 - 69 years old		1		1	
70 years or older	_____	1.07 (1.06-1.011)		1.07 (1.04-1.11)	
**Education**					
None/Incomplete fundamental education	_____	1.00 (0.96-1.03)		1.00 (0.97-1.04)	
Fundamental education	_____	1.00 (0.97-1.04)	0.12	1.01 (0.97-1.04)	0.16
Secondary education/ Incomplete	_____	1.00 (0.96-1.04)		1.01 (0.97-1.05)	
Undergraduate education	_____	1		1	
No Information	_____	0.96 (0.95-1.00)		0.97 (0.96-1.01)	
**Level 2 (Aggregated by FU)**					
**Density of Oncologist**					
5.72-28.24	_____	_____	_____	1.54 (1.41-1.68)	0.001*
29.56-40.92	_____	_____	_____	1.14 (1.10-1.18)	
41.23-60.10	_____	_____	_____	1	
**Density of Coloproctologist**					
0.00-1.06	_____	_____	_____	1.27 (1.19-1.35)	0.001*
1.40-1.87	_____	_____	_____	1.16 (1.11-1.21)	
1.99-10.41	_____	_____	_____	1	
**Density of Oncology Services**					
1.31-5.34	_____	_____	_____	1.08 (1.00-1.16)	0.001*
5.26-7.72	_____	_____	_____	1.10 (1.07-1.12)	
7.81-14.34	_____	_____	_____	1	
**Fixed effects**					
Intercept<br>(CI 95%)	-0.475<br>(-0.55- -0.39)	0.455<br>(0.31-0.42)		0.413<br>(0.38-0.49)	
**Random effects**					
Variance<br>(CI 95%)	0.0368<br>(0.020-0.065)	0.0292<br>(0.016-0.052)		0.0249<br>(0.036-0.046)	
LR Test<br>(x^2. p-value)	1031.28<br>(<0.001)	839.24<br>(<0.001)		767.71<br>(<0.001)	

PR: Prevalence ratio adjusted by the multilevel model with random intercept; CI: Confidence interval; p: Wald's test; * *p trend* ≤ 0.05; SP: São Paulo.

Model 1: Statistical model with inclusion of individual level variables; **Model 2:** Statistical model with inclusion of variables of individual level and contextual level per FU.

Table S2 - Multilevel analysis among individual and contextual variables for missing colorectal cancer stage in individuals aged 18 to 99 years old in the period from 2010 to 2019. Brazil, according to place residence, except SP (n = 103,367).

	Advanced Stage Of Colorectal Cancer n	%	PR	CI (95%)	p*
**Individual Variables**					
**Marital Status**					
Unmarried	14.494	62.9	1.16	1.13-1.19	<0.001*
Married	22.724	68.0	1.00	-	
No Information	8.110	64.0	1.12	1.09-1.15	
**Tobacco Smoking**					
Never	16.988	65.8	1.00	-	
Yes	12.983	67.9	1.03	1.02-1.05	<0.001*
No Information	15.357	63.6	0.97	0.95-0.98	
**Alcohol consumption**					
Never	18.005	65.0	1.00	-	
Yes	10.154	70.4	1.08	1.07-1.10	< 0.001*
No Information	17.169	63.7	0.97	0.97-0.99	
**Source of Referral**					
Public (SUS)	29.754	66.4	1.04	1.02-1.05	< 0.001*
Private/Health Insurance	6.257	64.0	1.00	-	
No Information	9.317	64.3	1.00	0.98-1.02	
**Socioeconomic contextual variables**					
**Percapita Income**					
360.34-749.69	21.155	64.8	1.28	1.12-1.46	
762.52-1039.30	23.759	66.3	1.23	1.07-1.40	<0.001*
1084.46-1715.11	414	72.5	1.00	-	
**Aging Rate**					
3.66-4.53	21.773	65.7	1.00	-	
4.75-8.83	1.956	61.7	1.12	1.07-1.17	<0.01*
9.07-26.09	21.599	65.9	0.99	0.97-1.01	
**Health service offer contextual variables**					
**Density of Family Doctor (per 100,000 inhabitants)**					
6.44-11.51	11.989	64.7	1.02	0.99-1.05	<0.001*
12.75-17.44	15.364	66.7	0.96	0.94-0.98	
17.80-33.96	17.975	65.4	1.00	-	
**Health Insurance Plan Density (per 100,000 inhabitants)**					
5.05-22.84	22.143	65.3	1.00	-	
23.61 -31.87	17.472	66.3	0.98	0.97-0.98	<0.002*
36.65-41.98	5.173	65.1	0.99	0.96-1.01	
**Rectosigmoidoscopy Exams (per 100,000 inhabitants)**					
0.00-14.68	21.469	66.7	1.02	1.01-1.04	<0.001*
18.60-39.98	12.695	64.5	0.99	0.98-1.01	
40.77-120.68	11.164	64.8	1.00	-	

PR: Estimated Prevalence Ratio by Robust Poisson Model; CI: Confidence interval; p: Wald’s test; *Statistically significant; SP: São Paulo.

Table S3 - Prevalence and unadjusted prevalence ratios for advanced stage colorectal cancer, according to individual characteristics and contextual variables. Brazil, by place residence, excluding SP (n = 69,047).
